# Species identity and diversity effects on invasion resistance of tropical freshwater plant communities

**DOI:** 10.1038/s41598-020-62660-1

**Published:** 2020-03-27

**Authors:** Antonella Petruzzella, Tauany A. da S. S. R. Rodrigues, Casper H. A. van Leeuwen, Francisco de Assis Esteves, Marcos Paulo Figueiredo-Barros, Elisabeth S. Bakker

**Affiliations:** 1 Department of Aquatic Ecology, Netherlands Institute of Ecology (NIOO-KNAW), Droevendaalsesteeg 10, 6708 PB Wageningen, The Netherlands; 2 Laboratório de Limnologia, Departamento de Ecologia, Instituto de Biologia, Universidade Federal do Rio de Janeiro (UFRJ), Av. Carlos Chagas Filho 373, 21 941-902, Cidade Universitária, Rio de Janeiro, Brazil; 3 Laboratório Integrado de Ecologia Aquática, Núcleo em Ecologia e Desenvolvimento Sócio-Ambiental de Macaé (NUPEM/UFRJ), Av. São José Barreto 764, 27 965-045, São José do Barreto, Macaé, Rio de Janeiro, Brazil

**Keywords:** Community ecology, Freshwater ecology, Invasive species

## Abstract

Biotic resistance mediated by native plant diversity has long been hypothesized to reduce the success of invading plant species in terrestrial systems in temperate regions. However, still little is known about the mechanisms driving invasion patterns in other biomes or latitudes. We help to fill this gap by investigating how native plant community presence and diversity, and the presence of native phylogenetically closely related species to an invader, would affect invader *Hydrilla verticillata* establishment success in tropical freshwater submerged plant communities. The presence of a native community suppressed the growth of *H. verticillata*, but did not prevent its colonisation. Invader growth was negatively affected by native plant productivity, but independent of native species richness and phylogenetic relatedness to the invader. Native plant production was not related to native species richness in our study. We show that resistance in these tropical aquatic submerged plant communities is mainly driven by the presence and biomass of a native community independent of native species diversity. Our study illustrates that resistance provided by these tropical freshwater submerged plant communities to invasive species contrasts to resistance described for other ecosystems. This emphasizes the need to include understudied systems when predicting patterns of species invasiveness and ecosystem invasibility across biomes.

## Introduction

Biological invasions represent a key component of human-induced global changes, and increasingly challenge the conservation and management of freshwater, marine and terrestrial ecosystems across the globe^1,2^. Although our knowledge on invasion ecology has increased considerably over the last decades, research has been strongly biased towards certain geographical regions and ecosystems^3–6^. Most of our understanding about the process, patterns and mechanisms of invasions emerges from studies performed in temperate terrestrial systems, whereas the impact on megadiverse tropical regions – that together harbor the greatest part of the Earth’s biodiversity – remains surprisingly understudied^3,5,7,8^. Similarly, freshwater systems, which are both hotspots of biodiversity and have been heavily impacted by human activities, are the least studied among ecosystem realms^3,9^. The lack of information is especially problematic because tropical systems are among the most threatened on earth, and freshwater ecosystems show the strongest biodiversity decline^10–12^. Understanding the mechanisms underlying the success or failure of invasions is essential for possible prevention, control or management of invasive species.

Studies have suggested that tropical systems may be less susceptible to invasions than temperate systems^13,14^. An important rationale for this idea is that the highly species-rich native communities generally found in tropical systems, may resist invasions through biotic processes such as competition^15^. The biotic resistance hypothesis predicts that, during alien plant invasion, more diverse communities are less susceptible to invasions than less diverse communities^16^. At small spatial scales, plant diversity may provide resistance through different niche-based mechanisms. For example, complementarity among multiple species (or functional groups) with non-overlapping resource use strategies could lead to a better use of available resources, leaving less resources available for a potential invader (the complementarity effect)^17,18^. Higher native diversity could also provide biotic resistance by increasing the probability to have a better competitor or a more productive species present in a more diverse native community, a mechanism known as the sampling effect^19^. This hypothesis is largely supported in temperate regions where plant diversity has been experimentally manipulated^17,20,21^, but there is little empirical evidence in tropical freshwater systems^22,23^.

In line with the biotic resistance hypothesis, stronger resistance to alien species may be expected by phylogenetically more closely related native species. Phylogenetic relatedness of alien species to native species of a community has been used as a metric of niche overlap, and lead to the concept of “ecological similarity“ of species^24,25^. Darwin^26^ in *On the origin of species* already proposed that invaders more closely related to native species would be more ecologically similar and therefore have greater niche overlap, leading to greater competition for the same resources^25–27^. It is important to highlight that the biotic resistance hypothesis and the ecological similarity concept are not mutually exclusive and can work synergistically^28^.

The current research bias on biological invasions towards temperate terrestrial ecosystems limits accurate predictions on the establishment and impact of invasive species on tropical aquatic systems, and therefore also limits the development of the field of invasion ecology in general and our ability to draw robust generalizations across biomes^4^. Here, we address this knowledge gap by presenting the results of an experiment in which we manipulated species diversity and identity of tropical native submerged plant communities, and simulated an invasion by the highly invasive alien submerged rooted plant *Hydrilla verticillata* (L.f.) Royle. *Hydrilla verticillata* is spreading rapidly through tropical and non-tropical areas^29^ and has negative impacts on shipping, recreation activities, fisheries, hydropower generation and threatens the ecological integrity of many tropical freshwater ecosystems^30,31^. We focused on invader establishment success, here defined as the ability of plant fragments to colonise and grow. We hypothesized that invader establishment success would be (1) greatest in the absence of a native plant community, (2) lower in species-richer plant communities, (3) lowest in the presence of the phylogenetically closely related species *Egeria densa*.

## Results

### The presence of a native community

The presence of native plants did not decrease invader colonisation success, i.e. the ability of the invader having its roots attached in the sediment (Fisher’s exact test, odds ratio = 1.869, *p* = 0.503). *Hydrilla verticillata* had fragments with roots attached to the sediment in 5 out of 6 mesocosms with bare sediment (bare) (83.3% colonised), whereas 38 out of 42 planted treatments, i.e. 90.5% of the mesocosms, were successfully colonised even though a native plant community was present. However, the presence of native plants did suppress *H. verticillata* growth, measured as root biomass (53.5% decrease), shoot biomass (27.5% decrease), root:shoot ratio (42.7% decrease), and RGR (18.9% decrease) (Table 1).

**Table 1:** Results of General Linear Mixed effect models testing how four different growth parameters of the invader species *Hydrilla verticillata* depended on the presence of a native community, native species richness (data shown in Fig. 1) and the presence of the phylogenetically related species *Egeria densa*.

Fixed effects	Root DW			Shoot DW			Root:shoot			RGR		
	*d.f*.	*t*	*P*	*d.f*.	*t*	*P*	*d.f*.	*t*	*P*	*d.f*.	*t*	*P*
Presence of native community	41	−4.85	<**0.001**	41	−3.15	**0.003**	41	−3.63	<**0.001**	41	−3.34	**0.002**
Species richness (including bare sediment)	41	−3.56	<**0.001**	41	−2.31	**0.026**	41	−3.07	**0.004**	41	−2.23	**0.031**
Species richness (excluding bare sediment)	35	−1.40 (log)	0.171	35	−0.48	0.633	35	−1.20	0.238	35	−0.22	0.825
Presence of *Egeria densa*	41	−2.26	**0.029**	41	−1.51	0.139	41	−1.66 (log)	0.105	41	−1.24	0.221

Block is included as a random factor. Significant effects (p < 0.05) are in bold. (log) means the response variable was log-transformed. Root DW = Root dry weight, Shoot DW = Shoot dry weight, Root:shoot = Root:shoot ratio, RGR = Relative growth rate.

### Effects of native community diversity and biomass on biotic resistance

**Figure 1:**
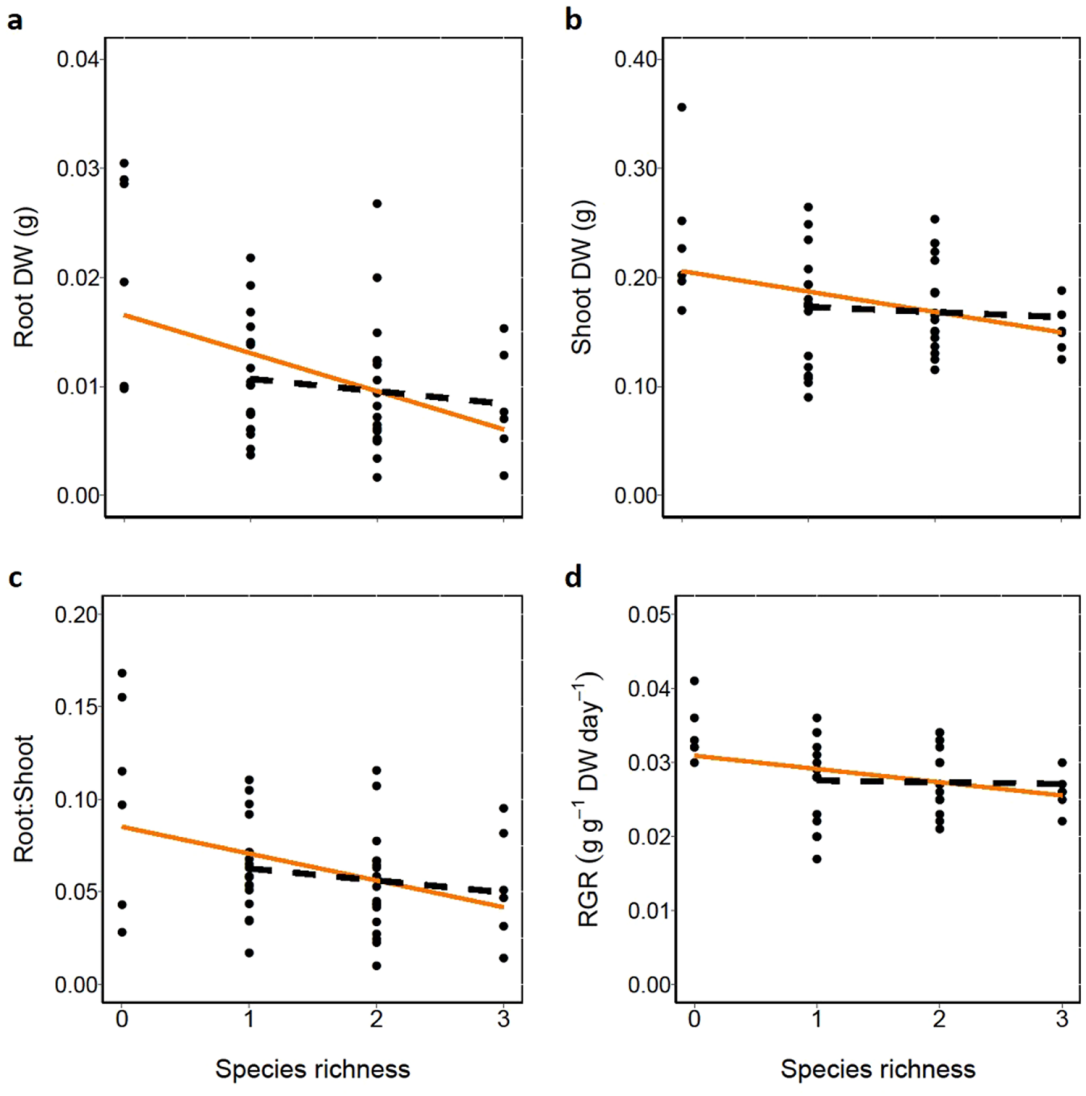
Effect of native plant species richness on the invader *Hydrilla verticillata* (**a**) root biomass (dry weight), (**b**) shoot biomass (dry weight), (**c**) root:shoot ratio, (**d**) relative growth rate (RGR). The orange solid lines are based on GLMMs that include the bare sediment treatments (0 species level) in the analysis, and are all statistically significant (Table 1). Black dashed lines are without including bare sediment (0 species level) in the analyses and are all not significant (Table 1). Each dot represents a replicate.

**Figure 2:**
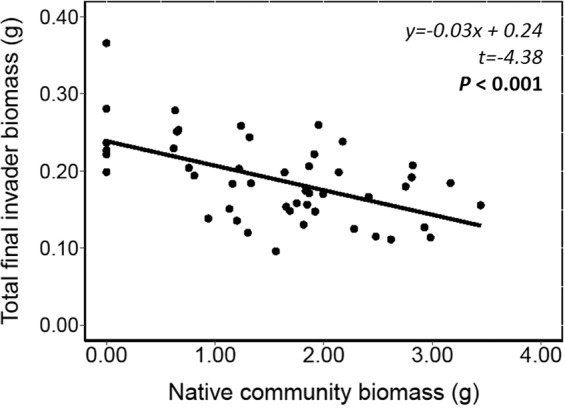
Relationship between the native community biomass and the total final biomass DW (dry weight, g) of the invader *Hydrilla verticillata*.

Native species richness significantly decreased invader root and shoot biomass, root:shoot ratio and RGR (Table 1; Orange solid line - Fig. 1a–d). However, when we excluded the bare sediment treatment from our analysis there was no effect of species richness on growth of *H. verticillata* (Table 1; Black dashed line - Fig. 1a–d). Native community biomass affected total final invader biomass significantly negatively (Fig. 2, SE = 0.05), whereas native plant biomass was not related to native species richness (*t* = 0.44, *P* = 0.6613). Total final native community biomass for all treatments is shown in Table 2.

**Table 2:** Overview of the 8 treatments used in the mesocosm experiment and the final mean native community biomass dry weight (g) across different treatments.

Diversity treatment	Abbreviated community composition	Species	Native community biomass (g) Mean ± SD, n = 6
1	Bare	Bare sediment	0 ± 0
2	C	*Cabomba furcata*	0.69 ± 0.08
3	E	*Egeria densa*	2.86 ± 0.19
4	U	*Utricularia foliosa*	1.67 ± 0.57
5	CE	*Cabomba furcata* + *Egeria densa*	1.89 ± 0.18
6	CU	*Cabomba furcata* + *Utricularia foliosa*	1.26 ± 0.24
7	EU	*Egeria densa* + *Utricularia foliosa*	2.29 ± 0.60
8	CEU	*Cabomba furcata* + *Egeria densa* + *Utricularia foliosa*	1.88 ± 0.56

Bare = Bare sediment, C = *Cabomba furcata*, E = *Egeria densa*, U = *Utricularia foliosa*.

### Limited effect of phylogenetic closely related species

**Figure 3:**
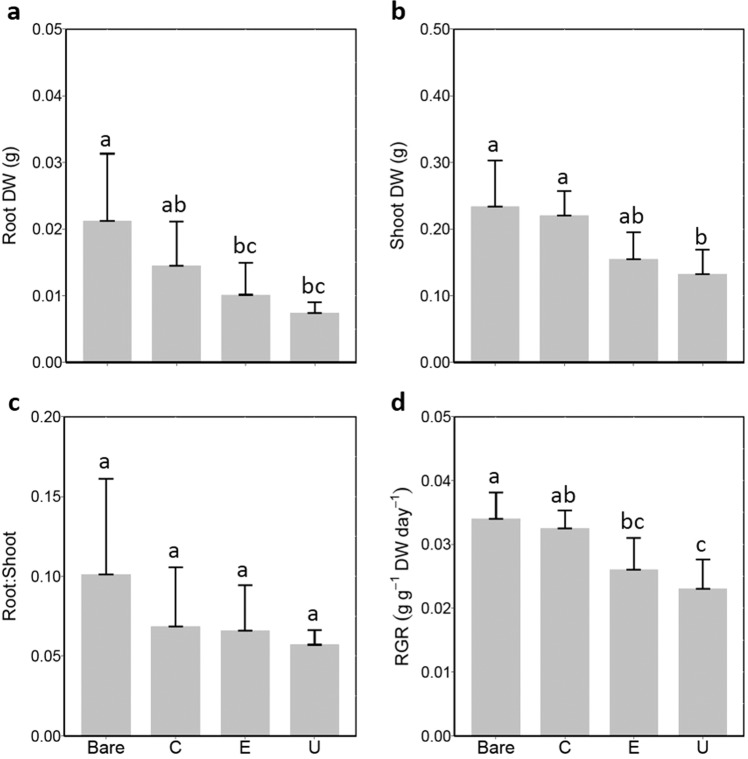
Effects of single native species on the invader *Hydrilla verticillata* (**a**) root biomass (dry weight), (**b**) shoot biomass (dry weight), (**c**) root:shoot ratio and (**d**) relative growth rate (RGR). Bare- Bare sediment, C- *Cabomba furcata*, E- *Egeria densa*, U- *Utricularia foliosa*. Different lowercase letters indicate statistically significant differences among treatments (Tukey’s HSD post hoc test). Significance levels were determined after Bonferroni’s correction for multiple testing (*P* < 0.008). The values are means ± standard error of the mean (n = 6).

The presence of the phylogenetically most closely related species *E. densa* in the native community significantly reduced invader root production, but shoot biomass, root:shoot ratio and RGR were not affected (Table 1). Native monocultures generally reduced invader growth, but the effect of *E. densa* monocultures was not stronger than of monocultures of *C. furcata* or *U. foliosa* (Fig. 3a–d). *Utricularia foliosa* monocultures consistently decreased *H. verticillata* root DW, shoot DW and RGR compared to the bare sediment treatment (Fig. 3a,b,d). However, these slightly negative effects did not significantly differ from those caused by the presence of *E. densa* monocultures for Root DW and RGR (Fig. 3a,d). *Hydrilla verticillata* was not affected by the presence of *C. furcata* at all. Root:shoot ratios of the invader were similar in all monocultures and the bare sediment treatment.

## Discussion

Competition from native species can decrease the establishment success of alien species^15^. Evidence for this mechanism originates predominantly from temperate terrestrial ecosystems, despite an increasing pressure of invasive species on megadiverse aquatic tropical systems^12^. We experimentally found that communities of native submerged tropical plants can have negative effects on the growth of the highly devastating invading species *H. verticillata*, but that this does not prevent its colonisation. Accordingly, we found that native community biomass negatively affected invader growth. Native productivity however, was not related to species richness in the community. Contrary to previous views, our results did not support the prediction that native species diversity provides greater resistance to plant invasions. The presence of the most closely related and ecologically similar native species (*E. densa*) also had only a limited effect on *H. verticillata* growth. Our study illustrates that resistance provided by these tropical native freshwater submerged plant communities to invasive species contrasts to resistance described for other ecosystems.

In our first hypothesis we predicted that invader establishment success, defined as the ability of invader fragments to colonise and grow, would be greatest in the absence of a native community. The proposed mechanism was that the presence of native plants should directly reduce resource availability for the invader in terms of nutrients or space, as observed for species in temperate systems^20^. However, the presence of the native community did not affect *H. verticillata* colonisation success (almost 90% of all mesocosms were colonised), despite that the native plants formed dense canopies in our experiment resulting from the uptake of available nutrients. We observed that invader fragments did not need to sink towards the sediment to attach their roots, as observed in other species of the family Hydrocharitaceae^20^. Instead, they started to produce long adventitious roots, which are fine and filiform^32^, when they were still floating on the water surface. The roots attached to the sediment after growing in between the shoots of the native plants (see Supplementary Fig. S1). Fragments of *H. verticillata* take more time to sink than fragments of other species^33^, but this ability to efficiently produce long roots while trapped in the native vegetation canopy gives them a competitive advantage to rapidly access nutrients in the sediment. Successful colonisation by *H. verticillata* is highly dependent on nutrient availability, as in a previous mesocosm experiment invader fragments could not colonise due to nutrient drawdown by the native plants^34^. Root production may be one of the factors that can help explain why this species is a strong coloniser in the field, as *H. verticillata* fragments were observed to colonise wetlands despite the presence of the native species *Vallisneria americana* Michaux^34^ and *Potamogeton pectinatus* (L.) Boemer or *Potamogeton gramineus* L.^35^. We therefore partially reject our first hypothesis that the presence of native vegetation would decrease *H. verticillata* colonisation, although it is supported regarding the ability of the invader to grow. The presence of a native plant community notably lowered root production of *H. verticillata* (53.5% decrease), so this could on the longer term at least slow down the colonisation process in shallow areas. This is found for a tropical invasive Poaceae species *Urochloa arrecta* (Hack. ex T. Durand & Schinz) Morrone & Zuloaga, which growth is limited by the native plant species but not sufficiently to completely repel the invader^23^. It is important to highlight that our experiment mimicked natural tropical shallow lake conditions and our experimental results must be interpreted with caution. *Hydrilla verticillata* has heavily invaded the Upper Parana River floodplain in Brazil which it can be found, for example, in many reservoirs and dams^31^. Studies attributed the success of *H. verticillata* colonisation in that region to its ability to colonise habitats that other native plant species are not able to tolerate such as relatively deep sites (4.0–7.3 m) and sites highly disturbed by waves^36,37^. Inorganic carbon is another factor that has been considered limiting the growth and productivity of submerged plant species^38,39^. Previous studies on *Cabomba* genus and *Utricularia* genus show that these plants strictly use CO_2_ as a source of inorganic carbon for photosynthesis whereas *H. verticillata* and *E. densa* can also use HCO_3_−^38,40,41^. The ability to use HCO_3_− ions associated with its high potential for invasion may be provided *H. verticillata* with a competitive advantage in alkaline waters such as the one presented by our experimental units (pH 7.9 ± 0.8) and the Jurubatiba lagoon (pH 7.8 ± 0.3).

Our second hypothesis follows Elton’s prediction that species-richer communities are more resistant to invasion than species poorer-communities^16^, as has been shown in a number of terrestrial and marine communities^17,42–44^. However, only few studies have investigated this principle in freshwater systems, and even fewer in tropical systems^20,22,23^. We did not find a relationship between native species richness and invader growth, although this depended on whether we included the bare sediment treatment in the analysis, as a treatment with zero species. If we did, we found a negative relationship between native species richness and growth parameters, whereas if we excluded the bare sediment treatment there was no relationship between native species richness and invader growth. Clearly, this indicates that this pattern is driven by the density of native plants, but independent of the native community diversity.

Some studies have acknowledged the potential that the densities and biomass of native plants may be more important for providing invasion resistance than native species richness *per se* in freshwater ecosystems^20,23,34,45–49^. An underlying mechanism of increased resistance to invader growth with increasing native species richness could be that communities with higher species richness have a higher productivity, and thus provide more resource competition through complementarity and/or sampling effects. Indeed, in our study invader growth was negatively affected by greater biomass production of native species, suggesting an underlying mechanism of increased resource competition. However, in our study, there was no relationship between native species richness and native biomass production, explaining the absence of increased resistance at higher native plant species richness. Therefore, we reject our second hypothesis.

*Egeria densa* and *H. verticillata* are species of the same family Hydrocharitaceae with similar growth form^31^. Although there is debate whether close relatives are more likely to have similar niches^50,51^, phylogenetic relatedness has been used as proxy for missing functional trait information, especially when using understudied tropical species. We expected lower invader establishment success in the presence of the closely related species *E. densa* (hypothesis 3). However, our study provides little support for the prediction that closely related species will compete more strongly than more distantly related species, hence we reject our third hypothesis. Therewith our study is in line with other studies partly supporting and partly rejecting this hypothesis over the last decade^24,52^. It is important to point out that members of the genera *Elodea*, *Lagarosiphon*, *Egeria* and *Hydrilla* include species that are all successful invaders outside of their indigenous ranges. However, although the species are similar in plant architecture, they vary in niche breadth and life history strategies. *Hydrilla verticillata* is recognised as probably the most invasive submerged macrophyte in the world^47,53^.

Despite that we did not find plant species effects, the *U. foliosa* monoculture provided greater resistance to *H. verticillata* growth compared to the bare sediment treatment. This effect of *U. foliosa* seemed to be greater than the effect of *E. densa*, even at lower biomass. This suggests that *U. foliosa* affected the invader by a different mechanism than by simply increasing overall biomass. This could be related to its dense, free submerged canopy structure inhibiting the invader from reaching the nutrient-rich sediment with its fragments: it took fragments of the invader four weeks to access the sediment in the presence of *U. foliosa*, and only two weeks in the bare sediment and *E. densa* monoculture treatments.

Our general understanding of biological invasions can be greatly advanced by coupling knowledge on invasions in all ecosystems worldwide. Our study highlighted that the ability of native freshwater submerged aquatic plant communities to resist invasive species may differ in some aspects from what is found in most other ecosystems^15,54^. These contrasts are important to understand when aiming to generalize patterns of species invasiveness and ecosystem invasibility across biomes. In contrast to most studies performed at small spatial scales in terrestrial ecosystems^15^, we found biomass to be more important for resistance to invasions than species richness per se. This is in line with the few previous studies on biotic resistance in freshwater ecosystems: even though some found a species richness effect on the invader success, most highlighted a crucial role of native species biomass for resistance^20,23,45^. Unlike studies conducted mostly in terrestrial systems, which often show a strong relationship between species (or functional) diversity and primary productivity, as observed in e.g. grasslands^55^, forests^56^ and a variety of other systems^57^, our experimental results showed that native productivity was not driven by native diversity. This suggests a mechanistic difference between aquatic and terrestrial ecosystems that warrants further investigation. This lack of positive diversity-productivity relationship was observed in other studies with submerged plant species^58,59^. A possible explanation for the general result of positive plant diversity-productivity relationships in terrestrial systems and different results for freshwater systems is may be the lack of complementarity effects among similar growth forms as submerged plants may in general be more similar in their resource use^60^. Even though we should be cautious about these considerations due to the low number of studies performed, this suggests the view that successful invasions are very context-dependent^61,62^. A system-specific understanding of community invasibility seems crucial for prediction and management of invasive species.

The intrinsic nature of tropical freshwater ecosystems, together with their importance and demand from a human perspective, may place them at greater risk of invasions than their temperate and terrestrial counterparts. Even though native plant biomass can decrease invader growth, it seems unable to repel establishment. Current intensification of transportation and trade in tropical areas increases the risk of new invader introductions in coming decades^12^. Together with great human-induced pressure, particularly overexploitation and habitat degradation and destruction which has been accelerating aquatic vegetation loss^63^, highlights the need to further study high susceptibility of tropical ecosystems to biological invasions. Furthermore, this study emphasizes the importance of conserving and restoring the native aquatic plant communities, especially after disturbances, because preserving native species and biomass increases the robustness of aquatic ecosystems to species invasions.

## Methods

We performed an outdoor mesocosm experiment in the northern region of the State of Rio de Janeiro at Núcleo em Ecologia e Desenvolvimento Sócio-Ambiental de Macaé (NUPEM), Federal University of Rio de Janeiro (UFRJ)- Macaé campus, Brazil (22°19′38.4″ S, 41°44'13.41″ W). The experiment ran for 10 weeks (71 days) from mid March to the end of May in 2017, during which the mean temperature in this region ranged from 22.7 °C to 23.8 °C (Instituto Brasileiro de Meteorologia, INMET).

### Plant material

We selected the highly invasive rooted submerged aquatic plant *H. verticillata* (Hydrocharitaceae) as invader. *Hydrilla verticillata* is native to Asia and Australia, but globally invades both tropical and temperate inland aquatic ecosystems due to its wide ecological amplitude, high growth rate, vegetative dispersal ability and low nutrient requirements for growth^31,64^. It can produce large amounts of vegetative propagules such as turions and fragments, which can float in the water column for days or weeks before settling and rooting^34,53^. Once *H. verticillata* is established it can rapidly produce large quantities of biomass and displace native macrophyte species^31,53^.

**Figure 4:**
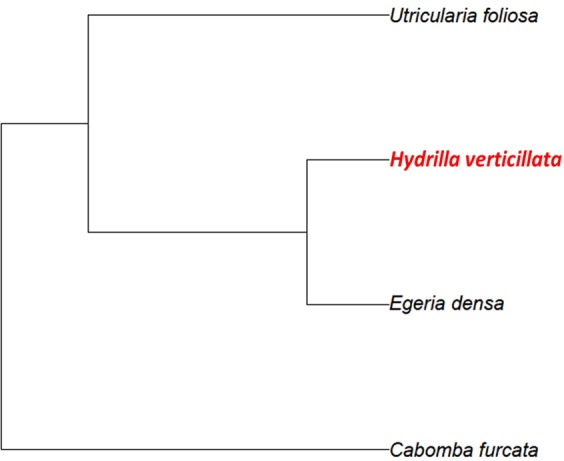
Phylogeny displaying phylogenetic relationships among the four species included in this study based on PhytoPhylo megaphylogeny^68^. The invader species *Hydrilla verticillata* is represented in red.

To test potential invasion resistance mediated by native plant communities on the establishment success of *H. verticillata* free floating fragments, here defined as the ability of plant fragments to colonise and grow, we established eight different native plant communities using combinations of three submerged macrophyte species: (1) the rooted submerged macrophyte *Cabomba furcata* Schult. & Schult. f. (Cabombaceae), (2) the rooted submerged species *Egeria densa* Planch. (Hydrocharitaceae), phylogenetically closely related to the invader, and (3) the non-rooted submerged species *Utricularia foliosa* L. (Lentibulariaceae) (Fig. 4). All three native species commonly co-occur and are widely distributed in tropical inland freshwater ecosystems of South America^41^.

The three native plant species also co-occur in the Jurubatiba lagoon, a freshwater coastal lagoon located in the Restinga de Jurubatiba National Park near the university campus (22°17′57.38″ S and 41°41′21.27″ W). However, because *C. furcata* and *E. densa* densities were low in the lagoon at the time of the experiment we obtained native plant material from three different locations in Rio de Janeiro State: *C. furcata* from São João River (22°34′54.9″ S, 42°18′1.04″ W), *E. densa* from Juturnaíba lagoon (22°36′47.9″ S, 42°16′30.03″ W) and *U. foliosa* from Jurubatiba lagoon. The invader *H. verticillata* was acquired from a commercial plant trader in Rio de Janeiro, Brazil (Flora Aquática). All four plant species were cultivated at the same growth conditions in 310 L water tanks (0.55 m height and 0.75 m diameter, two tanks per species). Each tank was filled with a layer of fertile substrate especially sold for plants in aquaria (7.5 kg/Mega fértil – Box Reef, organic matter content = 16%), a layer of washed sand (30 kg, ≤ 1.0 mm grain size, organic matter content = 0.5%), and water collected from Jurubatiba lagoon (mean ± SD, *n* = 6 water samples, 3.6 ± 0.4 µg L^−1^ P-PO_4_; 91.8 ± 12 µg L^−1^ N-NO_3_; 0.3 ± 0.8 µg L^−1^ N-NO_2_; 19.6 ± 31.6 µg L^−1^ N-NH_4_; pH 7.8 ± 0.3). After at least two weeks of cultivation, 183 non-rooted apical fragments without lateral branches were collected randomly from the six cultivation tanks for each of the native species. For *U. foliosa* the entire plant was collected instead of only the apical part. The plant fragments were cut to be 15 cm long and washed in running tap water to remove any material attached. Of the 183 fragments of each native species, 15 were randomly selected, dried to a constant mass at 60 °C for at least 48 h, and individually weighed for initial biomass measurements. The remaining 168 fragments were used in the experiment.

### Experimental set up

We performed a mesocosm experiment with 48 white plastic buckets (20 L, 36.7 cm height and 26 cm diameter) filled with a bottom layer of substrate for planted aquaria (Mega fértil – Box Reef, 1 cm/400 g) with a top layer of washed sand (5 cm deep, 4 kg sand per bucket). All mesocosms were placed outdoor in a covered area at NUPEM (see Supplementary Fig. S2). We filled all mesocosms with water from Jurubatiba lagoon. Water levels in the mesocosms were maintained constant during the experiment by refilling with lagoon water once a week to compensate for evapotranspiration. The physical and chemical parameters of the water were measured weekly in all mesocosms and the growing conditions were found to be suitable for the plants (mean values at daytime throughout the experiment, mean ± SD, n = 528: water temperature 23.4 ± 1.6 °C, dissolved oxygen concentration 8.8 ± 1.2 mg L^−1^, pH 7.9 ± 0.8, n = 288: alkalinity 478.1 ± 126.7 mEq L^−1^, turbidity 1.8 ± 4.2 NTU).

To test possible effects of native plant diversity and identity on the invasion potential of *H. verticillata* we established four levels of native plant species richness: zero species (bare sediment), one species (monocultures), and mixtures of two and three species (Table 2). The in total eight treatments were applied in a full factorial design, each replicated six times, using a randomized complete block design resulting in 48 mesocosms. Initial native plant density in the mesocosms was kept constant across treatments at 12 plant fragments per mesocosm, which is equivalent to 240 plants m^−2^ with the exception of the bare sediment treatment where the native plants were absent. Thus, mesocosms with two species contained six individuals of each species, whereas those with three species contained four individuals of each species. Such shoot densities are within the range of the shoot densities of submerged macrophyte communities in natural conditions^65^. The plant fragments were planted 5 cm deep in the sediment.

After the native plant community was allowed to establish in the mesocosms (determined by the growth of new shoots of at least one individual of each species, which took two weeks), we introduced two *H. verticillata* fragments per mesocosm. Each fragment consisted of a 15 cm long shoot with apical tip, but no lateral branches, to minimize variation in the size of the initial shoot material. This number of fragments is considered to represent medium propagule pressure^65^. We selected fragments with an apical tip, because these have higher regeneration and colonisation abilities, and higher growth rates than fragments without apical tips^66^. Invader fragments were not planted in the sediment but were dropped into the mesocosms simulating how alien species arrive in a new area where a native community is already established^67^. To determine the introduced biomass in dry weight (DW), fifteen *H. verticillata* fragments were randomly selected, dried to a constant mass at 60 °C for at least 48 h, and individually weighed.

### Plant harvest and data collection

We defined invader establishment success in terms of the ability of the fragments to colonise and grow. *Hydrilla verticillata* successful colonisation was defined as at least one of the introduced fragments having its roots attached in the sediment. At the end of the experiment, i.e. eight weeks (57 days) after the introduction of the invader fragments, we censused the mesocosms to check whether they were successfully colonised or not. *Hydrilla verticillata* growth was defined as biomass increase (DW, g). After the census, the invader was harvested, separated in root and shoot aiming to verify biomass allocation, dried to a constant mass at 60° for at least 48 h, and weighed. We determined *H. verticillata* total root and total shoot DW by summing the values from both introduced fragments. From these values we calculated the root:shoot ratio and relative growth rate (RGR). The RGR was calculated considering the total biomass of the invader (including roots plus shoots from both fragments) in the mesocosms as follows:

$${\rm{RGR}}=(\mathrm{ln}\,{W}_{{\rm{f}}}-\,\mathrm{ln}\,{W}_{{\rm{i}}})/{{\rm{t}}}_{{\rm{days}}}$$

where *W*_f_ = final DW, *W*_i_ = initial DW and t_days_ = time in days. At the end of the experiment, the native plant community biomass was harvested, sorted by species, dried to a constant mass at 60° for at least 48 h, and weighed.

### Phylogenetic tree construction

In order to show the phylogenetic relatedness among the study species we constructed a phylogenetic tree using the updated megaphylogeny of vascular plants (PhytoPhylo) as a backbone^68^. This megaphylogeny was constructed based on seven gene regions (i.e., 18S rDNA, 26S rDNA, ITS, *matK, rbcL, atpB*, and *trnL-F*), which include both slowly and quickly evolving regions. The time scale for the phylogeny was based on 39 fossil calibrations. Both minimum and maximum age constraints were utilized for each fossil calibration. We used S.PhyloMaker package in R developed by Qian and Jin^68^.

## Data analyses

We analyzed the colonisation success of *H. verticillata* fragments in the experimental mesocosms by comparing the proportion of colonised mesocosms between the bare sediment and planted treatments in a Fisher’s exact test. We fitted multiple General Linear Mixed-effects Models (GLMMs) to test how four growth parameters (i.e. root DW, shoot DW, root:shoot ratio and RGR) of the invader depended on the presence of the native community, native species richness (analyzed both including and excluding the bare sediment treatment, i.e. 0 species richness level from the analysis), and the presence of the phylogenetically closely related species *E. densa*, using the *lme* function in the R package *nlme*^69^. We used Tukey’s HSD multiple comparison test to compare means of species identity effect by each single native species. In two separate GLMMs we tested the effects of total native community biomass on total final invader biomass and native species richness on native community biomass. Block (the 6 replicates) was included as random factor in all models. Normality of model residuals, homoscedasticity and the influence of possible outliers were checked by visually inspecting plots of residual versus fitted values and quantile-quantile plots of model residuals. Response variables were log-transformed when necessary. All statistical analyses and graphics were performed in R^70^.

## Supplementary information

Supplementary Information.

## Data Availability

Data will be available in Dryad Digital Repository.
