# Supplementary material for: Species identity and diversity effects on invasion resistance of tropical freshwater plant communities
Source: Sci Rep. 2020 Mar 27;10:5626. doi: 10.1038/s41598-020-62660-1 (PMC7101304; doi:10.1038/s41598-020-62660-1)
Supplement: Supplementary file 1 — Supplementary Information. [file 41598_2020_62660_MOESM1_ESM.docx]

**Supplementary Information**

**Species identity and diversity effects on invasion resistance of tropical freshwater plant communities**

Antonella Petruzzella^1*^, Tauany A. da S. S. R. Rodrigues^2^, Casper H. A. van Leeuwen^1^, Francisco de Assis Esteves^2,3^, Marcos Paulo Figueiredo-Barros^3^ and Elisabeth S. Bakker^1^

^*^Corresponding author: [a.petruzzella@nioo.knaw.nl](mailto:a.petruzzella@nioo.knaw.nl)

**Fig. S1:**  Photograph of *Hydrilla verticillata* fragments producing long adventitious roots, which are fine and filiform, when they were still floating on the surface and attached to the sediment among the shoots of the monoculture of the native submerged species *Cabomba furcata*.

**
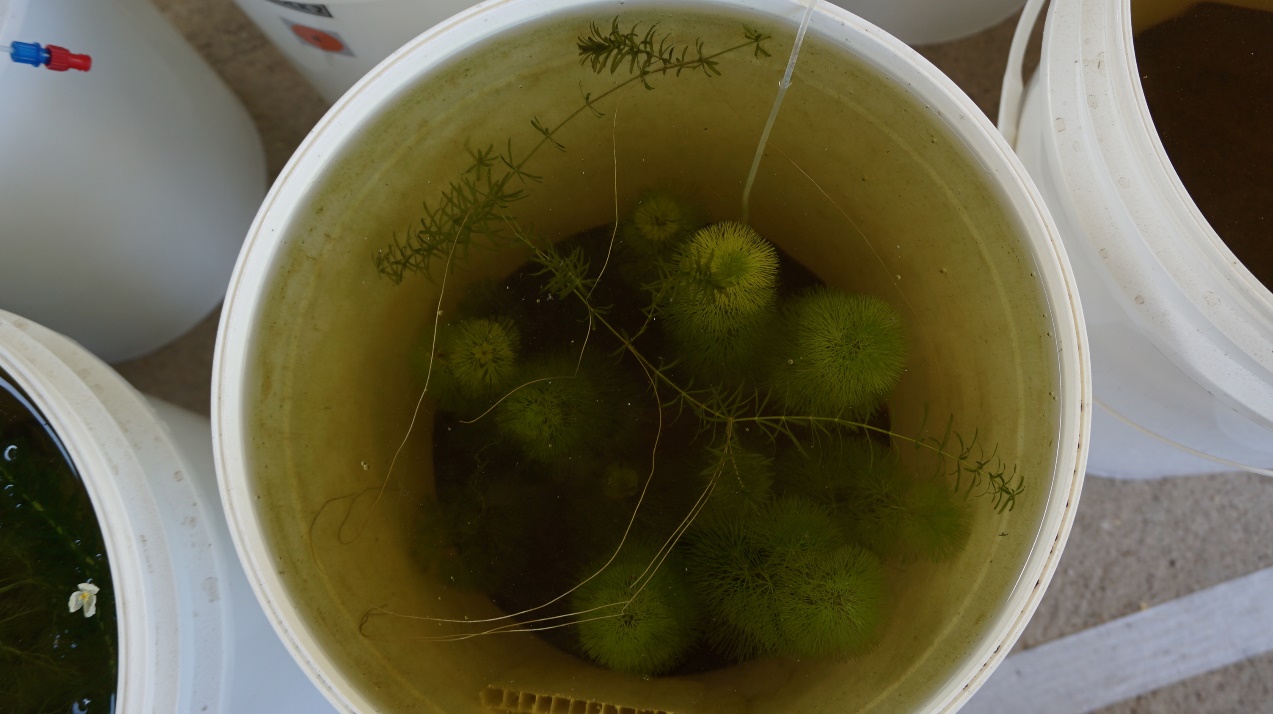
**

**Fig. S2:** Overview of the experiment (photo taken March 2017)

**
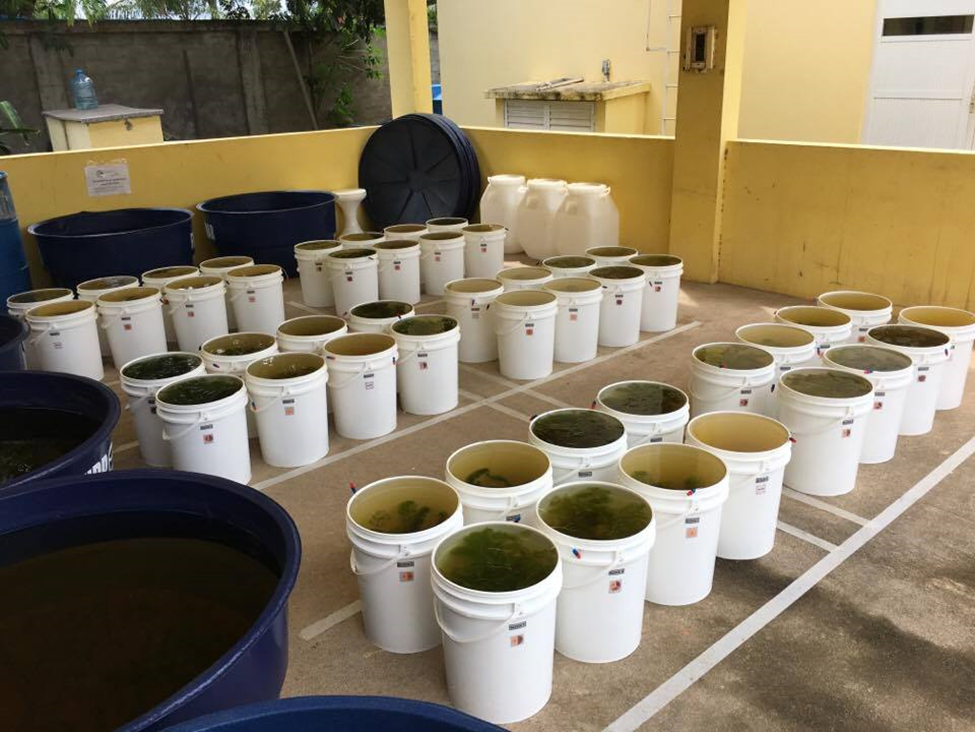
**
